# A review of the circuit-level and cellular mechanisms contributing to locomotor acceleration in the marine mollusk *Clione limacina*

**DOI:** 10.3389/fnins.2022.1072974

**Published:** 2022-12-22

**Authors:** Thomas J. Pirtle

**Affiliations:** Department of Biology, The College of Idaho, Caldwell, ID, United States

**Keywords:** *Clione*, CPG, locomotion, modulation, HCN, statocyst function, feeding behavior, neural circuit

## Abstract

The pteropod mollusk, *Clione limacina*, is a useful model system for understanding the neural basis of behavior. Of particular interest are the unique swimming behavior and neural circuitry that underlies this swimming behavior. The swimming system of *Clione* has been studied by two primary groups—one in Russia and one in the United States of America—for more than four decades. The neural circuitry, the cellular properties, and ion channels that create and change the swimming locomotor rhythm of *Clione*—particularly mechanisms that contribute to swimming acceleration—are presented in this review.

## Introduction

Many animals, by virtue of neural circuits called central pattern generators (CPGs) in their central nervous system, can create and control rhythmic movements (Delcomyn, 1980; Guertin, 2013; Bucher et al., 2015). These movements may be visceral in nature, controlling for example, heartbeat and digestive movement (Selverston et al., 1976; Arbas and Calabrese, 1984), or somatic, controlling body movements related to locomotion (Getting, 1981; Grillner and Wallén, 1985; Pearson and Wolf, 1987; Brodfuehrer et al., 1995; Arshavsky et al., 1998; Jing and Gillette, 1999; Thompson and Watson, 2005; Guertin, 2009; Fetcho and McLean, 2010; Zhong et al., 2012; Stein, 2018). This review summarizes the circuit-level and cellular properties of CPG interneurons of the marine pteropod mollusk, *Clione limacina*, that create and control the rhythmic movement of the animal’s wing-like parapodia. An added emphasis is on the mechanisms contributing to an increase in *Clione* locomotor speed.

**FIGURE 1:**
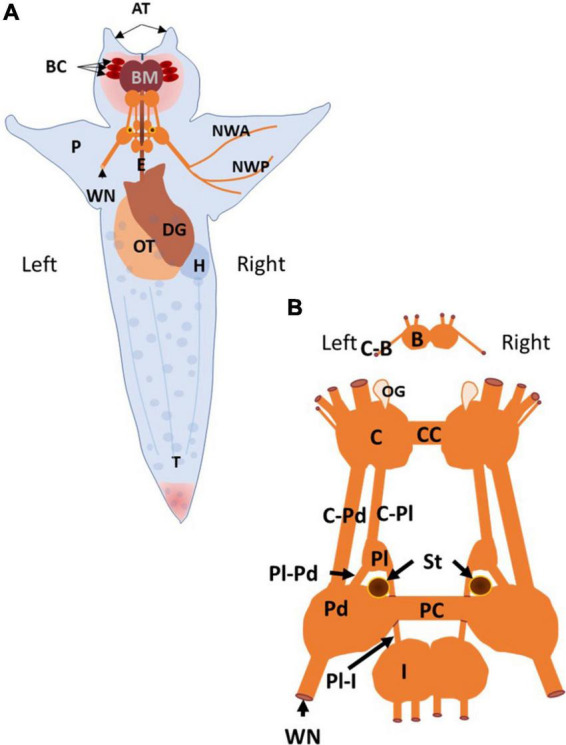
The anatomy of *Clione* and the organization of its central nervous system and related structures are illustrated. **(A)** The dorsal surface of *Clione* is depicted. *Clione* is typically engaged in slow, hovering swimming in which the long axis of the body is oriented perpendicularly to the ocean surface and ocean bottom with the head pointing upward and the tail pointing downward as depicted. The structures that are identified include retracted buccal cones (BC) and buccal mass (BM) found in the head and are important in prey capture and feeding. The buccal cones participate in prey capture and the buccal mass includes the radula and as set of chitinous hooks with associated musculature for extracting the prey, *Limacina helicina*, from its shell. Additionally, there are two laterally placed cephalic appendages, the anterior tentacles (AT) connected with the head of *Clione*. Between the anterior tentacles is slit-like mouth flanked by lips. *Limacina* is swallowed whole and passes down the esophagus (E) that is encircled by the central nervous system (CNS) composed of interconnected ganglia. Other organs identified include reproductive organs, the ovotestis (OT), digestive organs, the digestive gland (DG), and cardiovascular organs, the heart (H). Additionally, the major nerve innervating the wing-like parapodia (P), the wing nerve (WN) and its two primary branches (NWA and NWP) are identified. **(B)** The *Clione* CNS consists of paired ganglia that include the buccal ganglia (B), the cerebral ganglia (C), the pleural ganglia (Pl), the pedal ganglia (Pd), and intestinal ganglia (I). The buccal ganglia are connected to the cerebral ganglia *via* cerebro-buccal connectives (C-B; cut), the left and right cerebral ganglia are connected to each other by a commissure (CC) and connected to the ipsilateral pleural ganglia and ipsilateral pedal ganglia *via* the cerebro-pleural connective (C-Pl) and the cerebro-pedal connective (C-Pd), respectively. The pleural ganglia are connected to the ipsilateral pedal and intestinal ganglia *via* the pleuro-pedal and pleuro-intestinal connectives, respectively. The left and right pedal ganglia are connected to each other by the pedal commissure. The wing nerve is also connected to the pedal ganglia and carries afferent and efferent information related to the wing-like parapodia (P). Other structures identified include the sensory structures labeled OG, the olfactory organ, and ST, the statocysts (Wagner, 1885; Arshavsky et al., 1985a,b,c, 1990; Lalli and Gilmer, 1989; Hermans and Satterlie, 1992; Satterlie et al., 1995; Moroz et al., 2000; Zelenin and Panchin, 2000).

*Clione*, its major internal organs, and its central nervous system along with some peripheral nerves is shown in Figure 1. The *Clione* depicted in Figure 1A. is representative of the animal in its slow swimming mode in which the long axis of the body is oriented vertically in the water column with the head (containing the buccal cones, BC, and buccal mass, BM) pointing upward toward the water’s surface and tail (T) pointing directly downward toward the ocean bottom. Within the head are the prey capturing BC and feeding structures of the BM. There are three laterally placed BC on each side of *Clione*’s head that rapidly inflate to capture its prey, the shelled thecosome pteropod mollusk, *Limacina helicina*. The BM contains a feeding apparatus that includes chitinous hooks and radula, along with associated musculature. The hooks and radula grasp the *Limacina* and pull it from the shell to be swallowed. Other organs depicted in Figure 1 include organs of the reproductive system, the ovotestis (OT), organs of the digestive system, the esophagus (E) and digestive gland (DG), and the circulatory system, the heart (H; Wagner, 1885; Lalli and Gilmer, 1989). Because protraction of the prey capturing BC, and an increase in heartbeat occur with swimming acceleration, these structures are relevant to the discussion of the control of *Clione* swimming speed and are addressed in this review (Arshavsky et al., 1990; Norekian and Satterlie, 1995).

The central nervous system of *Clione*, shown in Figure 1B, like that of other gastropod mollusks, consists of pairs of connected ganglia that encircle the esophagus (E in Figure 1A) as a circumesophageal ring. From anterior to posterior, the ganglion of *Clione* includes paired buccal ganglia (B), cerebral ganglia (C), pleural ganglia (Pl), pedal ganglia (Pd), and intestinal ganglia (I). The right and left paired ganglia are connected by commissures, such as the right and left pedal ganglia that are connected *via* a pedal commissure (PC), or are fused together, such as the intestinal ganglia. There is no connecting commissure between the right and left pleural ganglia. Also shown in Figure 1B are the statocysts (St) that make up the vestibular or statomotor system of *Clione*, which contribute to the ability of *Clione* to orient its body within the water column and, during slow swimming, maintain the vertical body orientation as depicted in Figure 1B (Satterlie et al., 1985; Satterlie and Spencer, 1985; Panchin et al., 1995a). The wing nerve (WN) carries axons that innervate the parapodia and branches into two main branches, the anterior and posterior branches (NWA and NWP, respectively; Zelenin and Panchin, 2000).

**FIGURE 2:**
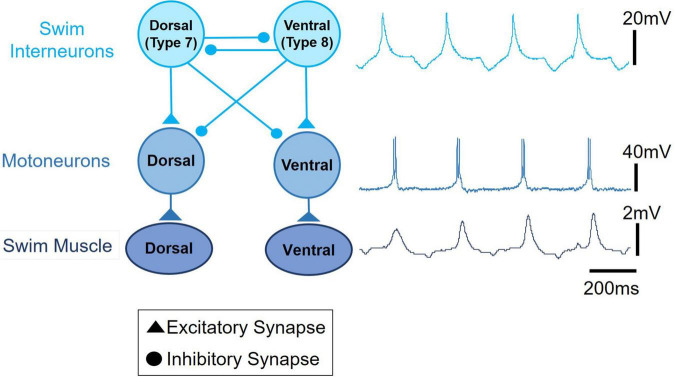
A schematic circuit representation of the *Clione* swim central pattern generator (swim CPG) network. The single broad action potential recorded from a dorsal swim interneuron, the burst of action potentials recorded from a synergistic dorsal small motoneuron, and the uncalibrated movement of the parapodia are displayed on the right next to the circuit diagram. Excitatory synaptic communication is shown by filled triangles and inhibitory synaptic communication is shown by closed circles. An important network feature of the *Clione* swim CPG is the reciprocal inhibitory synaptic communication between antagonistic groups of swim interneurons.

Swimming locomotion in *Clione* is produced by the dorsal-ventral movement of its parapodia (P in Figure 1A; also referred to as wings). Parapodial movement is controlled by swimming musculature that is in turn controlled by motoneurons whose rhythmic activity is produced and controlled by a circuit of swim interneurons making up the animal’s swim CPG (Arshavsky et al., 1985a,b,c; Satterlie, 1985; Satterlie and Spencer, 1985). A simple schematic diagram of the *Clione* swim CPG is illustrated in Figure 2. This diagram is a classic example of the half-center model first proposed by Brown (1911) and shows that the *Clione* swim CPG consists of two groups of swim interneurons. One group of swim interneurons controls the dorsal (or upstroke) movement of the parapodia, and the other group of swim interneurons controls the ventral (or downstroke) movement of the parapodia. Dorsal and ventral swim interneurons reciprocally inhibit each other so that when one group is active it simultaneously causes an inhibitory postsynaptic potential (IPSP) in the other group, thus inhibiting the other group. This reciprocal inhibition creates the alternating dorsal-ventral movement of the parapodia, and the duration of the IPSP is a principal variable that controls *Clione* swimming frequency (Arshavsky et al., 1993a; Satterlie et al., 2000). Furthermore, each swim interneuron produces a single broad action potential that conveys information to control synergistic motoneurons whose activity then controls the corresponding parapodial muscle contractions. Thus, there is a one-to-one correspondence between the activity of the interneuron, its synergistic motoneurons, and the parapodial muscle contraction produced by motoneuron innervation (Arshavsky et al., 1985b; Satterlie and Spencer, 1985).

**FIGURE 3:**
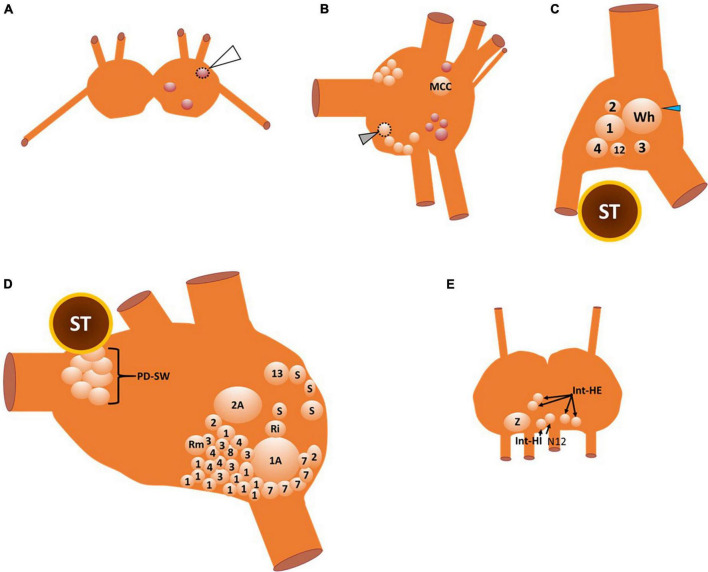
A map of the neuronal somas of the buccal ganglia **(A)**, right cerebral ganglion **(B)**, right pleural ganglion **(C)**, right pedal ganglion **(D)**, and intestinal ganglia **(E)**. Cells that are outlined by a dashed line are located on the ventral surface, otherwise all other somas are located on the dorsal surface. There are three neurons in the buccal ganglia **(A)** that are all GABAergic (see also Figure 4D). The one buccal ganglia soma that is outlined by the dotted line and indicated by the white arrowhead is located on the ventral surface of the buccal ganglia. The neurons of the cerebral ganglia **(B)** include the large serotonergic metacerebral cell that participates in feeding behaviors. The cluster of neuronal somas that occur posteromedial (including the ventral cell that is outlined with a dotted line and indicated by the gray arrowhead) and anteromedial near the cerebral commissure (cut) are serotonergic cells that evoke locomotor speed acceleration when stimulated. GABAergic neurons that are shown in red are important elements of the control of feeding behavior. In the right pleural ganglia **(C)** there are six neuronal somas (all located on the dorsal surface) identified that participate in swimming, whole-body withdrawal, and reproduction. The neuronal soma labeled 12 is the type 12 interneuron that is recruited into the *Clione* swim CPG during swim acceleration. Neuronal somas 1–4 participate in the whole-body withdrawal response that promotes wing and buccal cone retraction and inhibit swimming. The large soma labeled Wh and indicated by the blue arrowhead is the asymmetric pleural white cell that is only located in the right pleural ganglia. Wh participates in reproduction and egg laying. The neuronal somas of the right pedal ganglia **(D)** include interneurons that compose the *Clione* swim CPG, motoneurons, neurons that control the strength of parapodial movement, and neurons related to the parapodial (wing) withdrawal reflex. Odd numbers designate neurons that control the dorsal flexion of the parapodia while even numbers designate neurons that control the ventral flexion of the parapodia during swimming. The neurons labeled 7 and 8 are swim interneurons that compose the *Clione* swim CPG. The neurons labeled 1, 2, 3, and 4 are small motoneurons whose activity controls the slow-twitch swim muscle fibers within restricted innervation fields of the ipsilateral parapodia. Neurons 1A and 2A are large motoneurons that are also referred to as general excitor motoneurons (GEMN or GE) that innervate the entire expanse of the parapodia and communicate to both slow-twitch and fast-twitch swim muscle fibers in the parapodia. Neuron 13 is dopaminergic and controls swim inhibition. Neurons designated S, Ri, and Rm are sensory, interneuron, and motoneurons, respectively, that control the wing retraction reflex. Neuronal somas identified in the intestinal ganglia **(E)** control the heartbeat (all on the dorsal surface of the ganglia). These neurons include the intestinal heart excitor cells (Int-HE), Z cell (Z), and intestinal heart inhibitory cells (Int-HI). Axons of these neurons exit the median nerve, N12 (Arshavsky et al., 1985a,b,c, 1989, 1990; Satterlie, 1985, 1995a; Satterlie and Spencer, 1985; Norekyan, 1989; Huang and Satterlie, 1990; Norekian and Satterlie, 1996a; Malyshev et al., 2008).

The interneurons that compose the *Clione* swim CPG, the motoneurons that the CPG controls, and the neurons that contribute to the variability of locomotor activity and other related behaviors such as feeding and heartbeat regulation are illustrated in Figures 3A–E.

**FIGURE 4:**
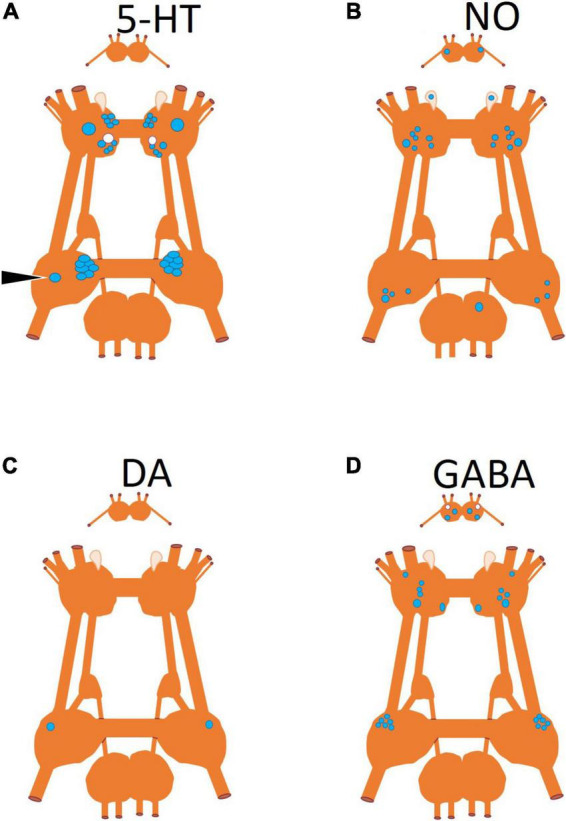
Neuronal somas mapped according to neurotransmitter produced by the neuron. **(A)** Serotonin (5-HT) identified by immunohistochemistry (Satterlie et al., 1995), **(B)** nitric oxide (NO) identified by nicotinamide adenine dinucleotide phosphate diaphorase (NADPH-d) histochemistry (Moroz et al., 2000), **(C)** dopamine identified through pharmacological antagonist blocking its physiological effect (Norekyan, 1989), and **(D)** gamma-aminobutyric acid (GABA) identified by immunohistochemistry (Norekian, 1999; Norekian and Malyshev, 2006). Somas that are blue are located on the dorsal surface of the ganglion and somas in white are located on the ventral surface of the ganglion.

The buccal ganglia are shown in Figure 3A. The three neuronal somas (colored red) in the buccal ganglia shown in Figure 3A are GABAergic (see also Figure 4D) and control aspects of feeding behavior. The GABAergic neuron indicated by the white arrowhead and outlined with a dashed line is located on the ventral surface of the buccal ganglion. Within the cerebral ganglia shown in Figure 3B, are serotonergic neurons (orange) that are important mediators of swim acceleration. One of these serotonergic neurons is found on the ventral surface of the cerebral ganglion (gray arrowhead and outlined with a dashed line). Additionally, shown in red and located laterally and caudally near the cerebro-pedal connective are four GABAergic neurons (see also Figure 4D) that participate in feeding behavior (Norekian, 1999). Within the pleural ganglia (Figure 3C) are neurons that include swim interneuron 12 that is recruited into the CPG circuit during swim acceleration (Arshavsky et al., 1985d,^1989^; Pirtle and Satterlie, 2006). Additionally, the neurons controlling whole body withdrawal and inhibition of swimming are in the pleural ganglia and include Pl-W neurons labeled 1–4 (Norekyan, 1989; Norekian and Satterlie, 1996a). The large asymmetrical white cell (blue arrowhead in Figure 3C) found in the right pleural ganglia mediates egg laying (Norekian and Satterlie, 2001). The neurons of the pedal ganglia include the swim interneurons composing the *Clione* swim CPG and the swim motoneurons controlled by the CPG circuit. Swim interneurons and the synergistic swim motoneurons that control the dorsal flexion of the parapodia are designated by odd numbers while the swim interneurons and the synergistic swim motoneurons that control the ventral flexion of the parapodia are designated by even numbers. Swim interneurons include the dorsal swim interneurons, 7, and ventral swim interneurons, 8 (see also Figure 2). There are swim motoneurons with relatively small soma called small motoneurons labeled 1, 2, 3, and 4. These small motoneurons have restricted innervation fields and control only the slow-twitch swim muscle of the parapodia. Two neurons labeled 1A and 2A are large motoneurons (referred to as general excitor motoneurons; GEMN or GE) that have an expansive innervation field covering the entire parapodia and control the contraction of both slow-twitch and fast-twitch swim muscle fibers (Arshavsky et al., 1985b; Satterlie and Spencer, 1985; Satterlie, 1993). Located in the medial part of the pedal ganglia near the pedal commissure are a cluster of serotonergic neurons called Pd-SW neurons that control the contractile force of parapodial slow-twitch muscle fibers (Satterlie, 1995b; Plyler and Satterlie, 2020). A single asymmetric serotonergic neuron in the left pedal ganglia is the heart excitor neuron (HE) that controls the heartbeat (Figure 4A; Arshavsky et al., 1990; Satterlie et al., 1995; Malyshev et al., 2008). The remaining neurons labeled in the pedal ganglion are the neurons that control the wing withdrawal reflex when the parapodia (wings) receive tactile stimulation. These neurons include sensory neurons (S), wing-retraction interneurons (Ri), and wing-retraction motoneurons (Rm; Huang and Satterlie, 1990). Interneuron 13 is dopaminergic and mediates swim inhibition (Norekyan, 1989). The neuronal somas in the intestinal ganglia shown in Figure 3E control the heart rate and include heart excitors that increase the heart rate, Int-HE and Z cell, and heart inhibitors, Int-HI, that decrease heart rate (Arshavsky et al., 1990; Malyshev et al., 2008).

In addition to the anatomical locations of neurons that control the behaviors of *Clione*, Figure 4 shows the neurotransmitters characteristic of these neurons (Figures 4A–D). The neurotransmitters, serotonin and GABA, were localized by immunohistochemistry (Norekian, 1999). Nitric oxide (NO) was localized using nicotinamide adenine dinucleotide phosphate diaphorase (NADPH-d) histochemistry (Moroz et al., 2000). The single pedal, neuron 13 (Figure 4) was identified as dopaminergic through pharmacological methods (Norekyan, 1989).

The neurons contained within the ganglia as described above were characterized by two different research groups—one in the United States of America (Satterlie and Norekian) and one in Russia (Arshavsky). Hence, the names of the same neurons are often different. Table 1 summarizes the names and other aspects of neurons to aid the reader in identifying the various names of neurons characterized by both Satterlie and Arshavsky research groups.

**TABLE 1:** Summary of neurons in this review: their alternative names and description.

	Satterlie and Norekian	Arshavsky	Description
Buccal	Bc-PIN	Bc-PIN	Arshavsky et al. (1993b); two paired symmetrical neurons
Cerebral	Metacerebral cell (MCC)	Metacerebral cell (MCC)	Arshavsky et al. (1993b) and Satterlie et al. (1995); two paired symmetrical neurons with somas of 90 μm; serotonergic
	Cr-SA	CPA1	Panchin et al. (1995b) and Satterlie et al. (1995); serotonergic with somas of 15–20 μm; located anteriorly near cerebral commissure; stimulation results in swim acceleration
	Cr-SP	CPB1	Panchin et al. (1995b) and Satterlie et al. (1995); serotonergic with somas of 15–30 μm; located posteriorly near cerebral commissure; stimulation results in swim acceleration
	Cr-SV		Norekian and Satterlie (1996a,2001); serotonergic; soma located on ventral surface of cerebral ganglia. Left Cr-SV soma is 50 μm and right Cr-SV soma is 30 μm; inhibit pleural withdrawal (Pl-W) neurons.
	Cr-T		Norekian (1995); exist as a bilateral pair (Cr-T1 and Cr-T2) on the ventral surface of the cerebral ganglia; somas are 80 μm for Cr-T1 and 60 μm for Cr-T2
	Cr-A	TenMN	Norekian and Satterlie (1993) and Arshavsky et al. (1993b); 50–100 μm depending on specific member of the Cr-A group; somas located on both dorsal and ventral surfaces of cerebral ganglia; motoneuron that triggers buccal cone eversion (protraction)
	Cr-B		Norekian and Satterlie (1993); soma diameters of 40–60 μm depending on the specific member of the Cr-B group; somas located on both dorsal and ventral surfaces of cerebral ganglia; motoneuron that triggers buccal cone withdrawal (retraction)
	Cr-Pc		Norekian and Satterlie (1995); somas 35 μm; symmetrical left-right pairs located in anterior cerebral ganglia
	Cr-BM		Norekian and Malyshev (2005); somas 25–30 μm; GABAergic
Pleural	Type 12 interneuron	Type 12 interneuron	Arshavsky et al. (1989); single neuronal soma in each of the pleural ganglia with somas of 30–40 μm
	Pl-W	Pl-W	Norekian and Satterlie (1996a); several neurons in the pleural ganglia compose the group with the majority being small (45–55 μm diameter somas) and one large with 150 μm diameter soma
	White cell	Pl-Wh	Norekian and Satterlie (2001); single asymmetric neuronal soma in the right pleural ganglion; becomes opaque with sexual maturation, involved in egg laying
Pedal	Up or dorsal swim interneuron	7	Arshavsky et al. (1985b), Satterlie (1985), and Panchin and Sadreyev (1997); compose the *Clione* swim CPG controlling the dorsal flexion of parapodia; 20–40 μm diameter soma; cholinergic
	Down or ventral swim interneuron	8	Arshavsky et al. (1985b), Satterlie (1985), and Panchin and Sadreyev (1997); compose the *Clione* swim CPG controlling the ventral flexion of parapodia; 20–40 μm diameter soma; glutaminergic
	Up/dorsal or down/ventral small motoneurons	1, 2, 3, and 4	Arshavsky et al. (1985b), Satterlie (1995a), and Satterlie and Courtney (2008); innervate limited areas of the parapodial slow-twitch swim musculature; somas with diameters of 20–40 μm; odd numbers control dorsal flexion of parapodia and even numbers control ventral flexion of parapodia; cholinergic
	Up/dorsal or down/ventral general excitor motoneurons	1A and 2A	Arshavsky et al. (1985b) and Satterlie (1995a); innervate the entire expanse of the parapodia and innervate both slow-twitch and fast-twitch swim muscle fibers; somas are 80 μm; odd numbers control dorsal flexion of parapodia and even numbers control ventral flexion of parapodia
	Pd-SW	Pd-SW	Plyler and Satterlie (2020); 5–9 somas with 20–80 μm diameter near the pedal commissure; serotonergic; innervates slow-twitch muscle fibers to increase the contractility of these muscle fibers
	HE	HE	Arshavsky et al. (1990) and Satterlie et al. (1995); asymmetric serotonergic neuron of left pedal ganglia; 30–40 μm diameter soma; excites ventricle
	S		Huang and Satterlie (1990); 30–40 μm diameter somas; sensory neuron mediating wing retraction reflex
	Ri		Huang and Satterlie (1990); 15–20 μm diameter somas; interneuron mediating wing retraction reflex
	Rm		Huang and Satterlie (1990); 30–40 μm diameter somas; motoneuron mediating wing retraction reflex
Intestinal	Int-HE	Int-HE	Malyshev et al. (1999); 50 μm diameter somas; excites both auricle and ventricle
	Int-HI		Arshavsky et al. (1990); 20–30 μm diameter somas; inhibits both auricle and ventricle
	Z cell		Malyshev et al. (2008); asymmetric neuron in left intestinal ganglia with soma (largest in left intestinal ganglia) of 150 μm diameter somas; excites auricle

**FIGURE 5:**
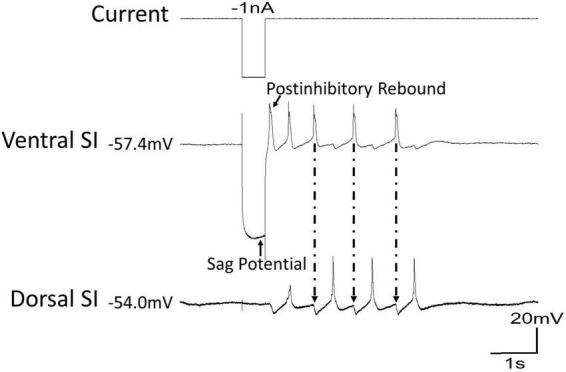
During a time of swim cessation, a hyperpolarizing current of –1 nA injected into the ventral swim interneuron of *Clione* evokes a sag potential during the hyperpolarization followed by a rebound in action potential activity creating a short bout of swimming. Arrows with dashed lines show the inhibitory postsynaptic potentials (IPSPs) in the dorsal swim interneuron produced by the action potentials of the ventral swim interneuron.

Two cellular properties of *Clione* swim interneurons that contribute to locomotor pattern generation are the sag potential and postinhibitory rebound (Pirtle and Satterlie, 2007, 2021; Pirtle et al., 2010). The sag potential, which relates to the current called I_h_ mediated by hyperpolarization-cyclic nucleotide-gated ion channels (HCN channels) will be addressed later in this review. Postinhibitory rebound is a depolarization that occurs following inhibition of swim interneurons. Thus, postinhibitory rebound plays a crucial role in locomotor pattern generation by allowing inhibited swim interneurons to quickly rebound to reach the threshold and produce an action potential (Satterlie, 1985; Arshavsky et al., 1993a; Satterlie et al., 2000; Pirtle and Satterlie, 2004, 2007). Figure 5 shows how postinhibitory rebound produced in a ventral swim interneuron by applying a−1 nA current injection can quickly bring about swimming in *Clione* during a state of swimming cessation. Following the −1 nA current injection, the ventral swim interneuron produces an action potential that in turn inhibits the dorsal interneurons. A simultaneous recording of membrane potential from the dorsal swim interneuron shows that it also generates a postinhibitory rebound-induced action potential that inhibits the ventral swim interneuron. This alternating pattern of activity continues as a brief bout of fictive swimming that lasts approximately 4 s.

Several molluscan species have contributed to our understanding of locomotor CPGs and their modulation. Among these are *Tritonia diomedea* (Getting, 1981), *Pleurobranchaea californica* (Jing and Gillette, 1999), *Aplysia brasiliana* (Gamkrelidze et al., 1995), *Melibe leonina* (Thompson and Watson, 2005), and *C. limacina* (Arshavsky et al., 1985a,b,c; Satterlie, 1985; Satterlie and Spencer, 1985). All these molluscan species are useful model systems for elucidating mechanisms of locomotor control because, compared to the more complex vertebrate animals, they all have simple neural circuits and large re-identifiable (i.e., neurons that can be identified in the same ganglion location between individual animals) neurons whose electrical properties can be studied in reduced semi-intact preparations. Additionally, mollusks show complex behaviors related to locomotion within an environmental context.

Advantages among the molluscan species used as a model for studying locomotor CPGs stem from their differences in ecological niche and related behaviors. *Clione* is unique among the related mollusks used in the study of locomotor CPGs for two major reasons. First, *Clione* occupies the midwater realm of the ocean where it keeps its position within the water column by virtually constantly swimming (Mackie and Mills, 1983; Arshavsky et al., 1985a; Mackie, 1985; Satterlie et al., 1985). *Clione* begins to sink if it ceases to swim, and it is hypothesized that *Clione* migrates up and down in the water column (Mackie, 1985) through the modulation of swimming behavior—*Clione* moves upward towards the surface of the water column by swimming and moves down the water column by inhibiting its swimming behavior (Arshavsky et al., 1992; Satterlie et al., 1985). Because *Clione* typically keeps its position within the water column by hovering locomotion, it is virtually in a constant state of motion. Thus, one advantage of *Clione* is that there is little need to activate the swim CPG interneurons to produce fictive swimming behavior by virtue of the animal being in a state of near-continuous swimming (Satterlie et al., 1985). Second, *Clione* can change its locomotor speed in response to feeding and predator avoidance (Arshavsky et al., 1985a). Thus, *Clione* is an invaluable model system for assessing the mechanisms that underlie locomotor speed change. Much of the research done on the *Clione* swim CPG has focused on mechanisms contributing to swim acceleration at both the circuit-level and cellular level within the swim CPG (Arshavsky et al., 1989; Kabotyanskii and Sakharov, 1991; Satterlie, 1991; Norekian and Satterlie, 1996a; Panchin et al., 1996; Satterlie and Norekian, 1996; Satterlie et al., 2000; Pirtle and Satterlie, 2021).

*Clione* is holoplanktonic and, as previously said, lives in the midwater where it keeps its position in the water column by engaging in active swimming (Lalli, 1967; Mackie and Mills, 1983; Mackie, 1985; Lalli and Gilmer, 1989). However, *Clione* can stop and restart locomotion depending on behavioral contexts. For example, *Clione* will accelerate swimming when hunting and when escaping potential predators (Arshavsky et al., 1985a,^1992^; Satterlie and Spencer, 1985). Similarly, tactile stimuli to the animal’s anterior region or parapodia elicit a behavioral response that consists of retraction of the head, the parapodia, and the tail leading to inhibition of swimming—a “whole-body withdrawal” response (Norekian and Satterlie, 1996a). Therefore, neural circuitry that controls *Clione* swimming behavior must be malleable to alter the locomotor speed.

Animals, including *Clione*, can accelerate locomotor speed by increasing the rate of locomotor appendage displacement, increasing the applied force of the locomotor appendage, or a combination of both increasing the rate of locomotor appendage displacement and increasing the applied force of the locomotor appendage (Satterlie, 1995a,b). Additionally, *Clione* can alter their locomotor speed through mechanical changes in their parapodia that include changing the stiffness of the parapodia and changing the angle of attack of the parapodia (Szymik and Satterlie, 2011, 2017). Stimulation of cerebral serotonergic neurons in *Clione* accelerates swimming locomotion at the circuit level through reconfiguration of the animal’s CPG and recruitment of more neurons.

Stimulation of serotonergic neurons found in the anterior and posterior portions of the cerebral ganglia (Cr-SA and Cr-SP neurons, respectively) and a serotonergic neuron on the ventral surface of the cerebral ganglia (Cr-SV) in *Clione* modulates swimming behavior by increasing locomotor speed through circuit level and cellular level mechanisms (Panchin et al., 1995b,^1996^; Satterlie, 1995b; Satterlie et al., 1995; Satterlie and Norekian, 1995, 1996). From a non-modulated state of slow swimming, serotonergic modulation can initiate swim acceleration in *Clione* by one of three general mechanisms: (1) by increasing parapodial contractility without a change in the frequency of parapodial movement (peripheral modulation only), (2) by increasing the frequency of parapodial movement without a change in parapodial contractility (central modulation only), or (3) by increasing both the parapodial contractility and by increasing the frequency of parapodial movement (both peripheral and central modulation; Satterlie and Norekian, 1996).

**FIGURE 6:**
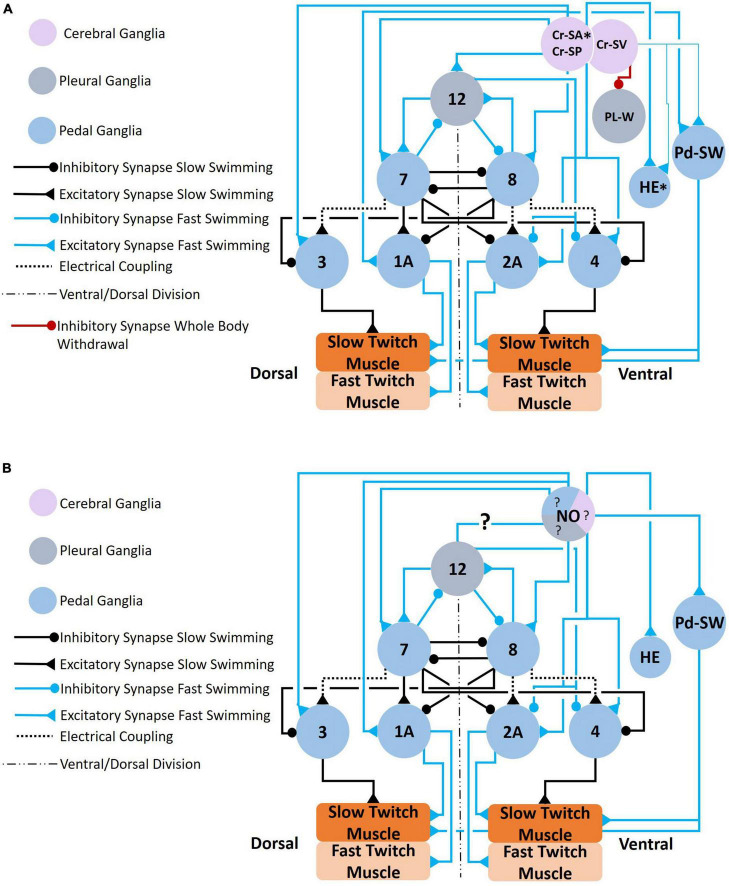
**(A)** Circuit mediating locomotor acceleration through the recruitment of neurons (blue connections). Three groups of cerebral serotonergic neurons, Cr-SA, Cr-SP, and Cr-SV, control recruitment of both interneurons and motoneurons in the swim system during swim acceleration. The Cr-SA and Cr-SP neurons (all on the dorsal surface of the cerebral ganglia) have overlapping functions—they recruit the type 12 interneuron into the *Clione* swim CPG, recruit large 1A and 2A (GEMNs), and recruit serotonergic neurons, Pd-SW neurons (that control force of contraction of slow-twitch swim muscle). The Cr-SA neurons are the only member of the Cr-SA/Cr-SP group to stimulate the asymmetric heart excitor (HE) neuron of the left pedal ganglia, which is indicated by the asterisk. The Cr-SV neuron is found on the ventral surface of the cerebral ganglia and weakly excite the HE and Pd-SW neurons as indicated by the thinner blue lines. The Cr-SV neuron also simultaneously inhibits the pleural withdrawal neurons, Pl-W, that participate in the competing whole-body withdrawal response. **(B)** Circuit mediating swim acceleration by NO. Nitrergic neurons are found in the cerebral (purple), pleural (gray), and pedal (blue) ganglia. However, it is not known which specific nitrergic neurons participate in swim acceleration. The responses of the elements of the swim system to NO were determined experimentally by bath application of NO-donors. The effect that NO has on the type 12 interneuron of the pleural ganglia has not been established (Norekian and Satterlie, 1996b,^2001^; Satterlie and Norekian, 1996; Moroz et al., 2000; Pirtle and Satterlie, 2021).

**FIGURE 7:**
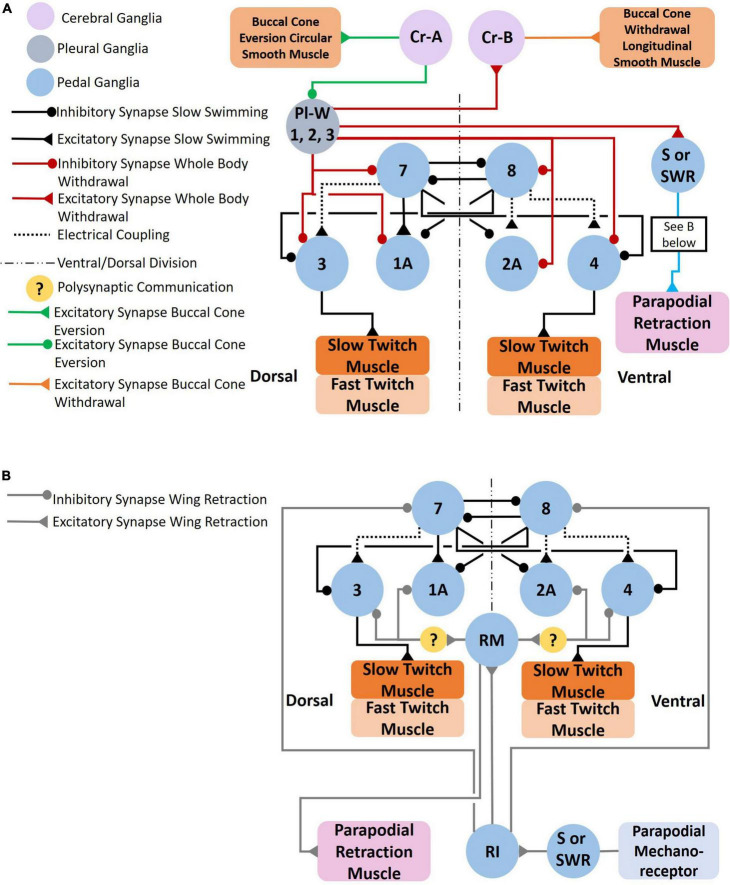
**(A)** Circuit mediating the *Clione* whole-body withdrawal response and buccal cone eversion and withdrawal. Stimulus to the head of *Clione* elicits the passive defensive withdrawal behavior through the activation of Pl-W neurons. Pl-W neurons in the pleural ganglia promote a passive defensive withdrawal behavior by inhibiting the swim system and promoting the withdrawal of buccal cones through the activation of Cr-B neurons that control the longitudinal smooth muscle of the buccal cones consequently leading to buccal cone withdrawal. The Pl-W neurons not only inhibit the swim CPG interneurons and swim motoneurons, but also work in concert with pedal neurons mediating the wing retraction reflex **(B)** by stimulating the wing mechanosensory neurons in the pedal ganglia (labeled S in Figure 3D) that mediate the wing retraction reflex. Furthermore, Pl-W neurons stimulate intestinal heart inhibitory neurons (Int-HI; see Figure 8) to inhibit the heartbeat. Feeding behavior overrides the withdrawal reflex. When feeding, Cr-A neurons simultaneously promote buccal cone eversion (see also Figures 9A, B) and inhibit Pl-W neurons (Norekyan, 1989; Huang and Satterlie, 1990; Norekian and Satterlie, 1996a; Malyshev et al., 2008).

**FIGURE 8:**
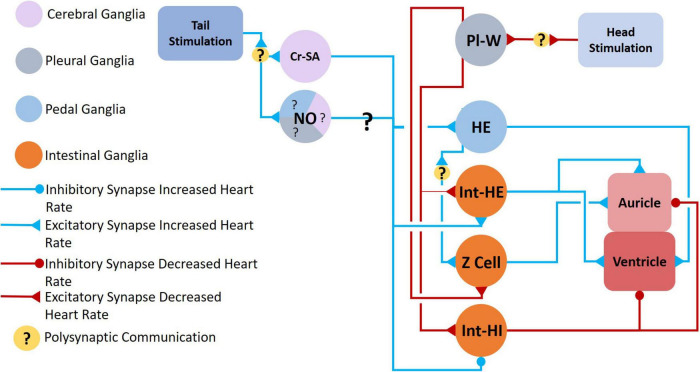
Neural circuit mediating changes in heartbeat. *Clione* heart rate increases during swim acceleration and is controlled by serotonergic Cr-SA neurons and NO, both of which also mediate swim acceleration. The exact nitrergic neurons involved is unknown (hence, the question marks—?—for NO). Tail stimulation accelerates swimming locomotion and increases heart rate through the excitation of serotonergic heart excitor (HE) neurons and neurons of the intestinal ganglia, Int-HE and Z cell. Head stimulation produces the opposite response—a decrease in heart rate—through stimulation of heart inhibitory neurons in the intestinal ganglia, Int-HI. Polysynaptic communication is indicated (Arshavsky et al., 1990; Satterlie and Norekian, 1995; Malyshev et al., 2008).

**FIGURE 9:**
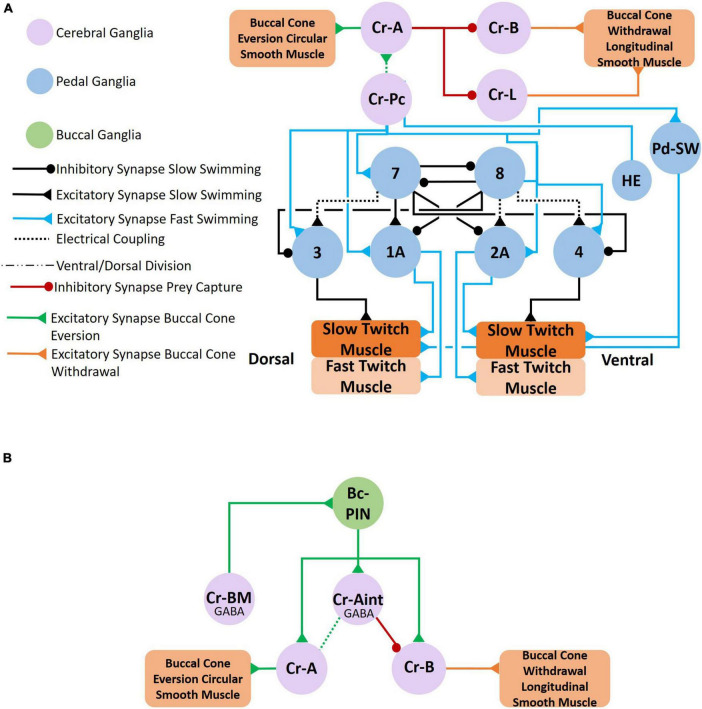
Feeding behavior is evoked when *Clione* encounters its prey, *Limacina helicina*. Feeding behavior involves the eversion of prey capture appendages—the buccal cones—and results in swim acceleration. **(A)** Cr-Pc (prey capture neurons) in the cerebral ganglia coordinate both feeding behavior and locomotor acceleration. First, Cr-Pc neurons activate Cr-A neurons through chemical synaptic communication and electrical coupling. The Cr-A neurons in turn activate circular smooth muscle in the buccal cones to facilitate buccal cone eversion during prey capture. Cr-Pc neurons also stimulate swim interneurons of the *Clione* swim CPG, stimulate motoneurons (including 1A and 2A motoneurons), Pd-SW neurons and the HE neuron. Thus, Cr-Pc neurons promote swim acceleration and increased heart rate, the later contributing to hydraulic inflation of the buccal cones during prey capture. In addition to promoting buccal cone eversion, Cr-A neurons simultaneously inhibit the competing buccal cone withdrawal by inhibiting Cr-B and Cr-L neurons that stimulate the buccal cone’s longitudinal smooth muscle fibers. **(B)** The cerebral GABAergic neurons, Cr-BM and Cr-Aint, mediate co-activation of motoneurons that control both buccal cone eversion and buccal cone withdrawal. Cr-BM excites the Bc-PIN interneuron of the buccal ganglia. The Bc-PIN in turn excites the Cr-Aint and simultaneously excites competing motoneurons, Cr-A motoneurons that control the buccal cone circular smooth muscle and promote buccal cone eversion and Cr-B motoneurons the control the buccal cone longitudinal smooth muscle and promote buccal cone withdrawal. Co-activation of Cr-A and Cr-B motoneurons occur during removal of the prey, *L. helicina*, from its shell (Arshavsky et al., 1993a,b; Norekian and Satterlie, 1993, 1995; Norekian, 1995; Norekian and Malyshev, 2005, 2006).

**FIGURE 10:**
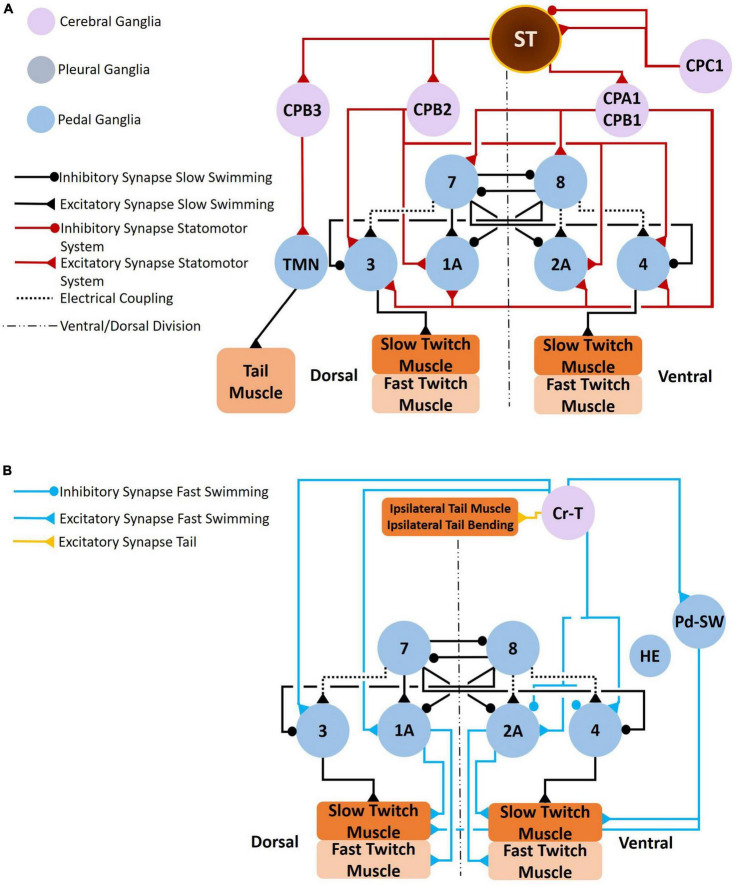
*Clione* postural responses, which involve tail movements, change in response to swim acceleration and feeding behavior. The circuit that coordinate tail movements to swim acceleration are illustrated in **A,B**. As shown in **A**, the statocyst receives input from both gravity and CPC1 neurons. CPC1 neurons have differential affects on the statocyst receptor cells (SRCs) depending on SRCs innervated. The SRCs of the statocysts send excitatory signals to CPA1/CPA2 neurons, CPB2 neurons, and CPB3 neurons. The CPA1/CPB1 neurons stimulate interneurons of the *Clione* locomotor CPG and stimulate swim motoneurons (including the large 1A and 2A motoneurons). The CPB2 neurons stimulate only motoneurons (including the large 1A and 2A motoneurons). Thus, the SRCs promote swim acceleration. Furthermore, SRCs stimulate CPB3 neurons that control tail motoneurons (TMN) that in turn control the bending of the *Clione* tail (T in Figure 1A). Tail bending acts like a rudder to steer *Clione* in circular and spiraling movements that are characteristic of feeding behavior. Coordinated tail bending is also achieved through the activation of Cr-T neurons **(B)**. Cr-T neurons stimulate Pd-SW neurons to enhance the force of parapodial contraction, stimulate motoneurons (including the large 1A and 2A motoneurons), and stimulate the tail musculature to bend the tail in a coordinated fashion (Norekian, 1995; Panchin et al., 1995a,b,c).

Central and peripheral serotonergic modulation of *Clione* locomotor activity involves changes at the circuit and cellular levels. At the circuit level, serotonin reconfigures the CPG circuit by recruiting several neurons that include 1A and 2A motoneurons, also called general excitor motoneurons (GEMN), in the pedal ganglia, pedal serotonergic neurons (Pd-SW) in the pedal ganglia, type 12 interneurons in the pleural ganglia, and type 8e interneurons of the pedal ganglion (Satterlie and Norekian, 1996; Arshavsky et al., 1998). These circuit-level changes that recruit neurons are shown in Figures 6–10.

## Circuit-level reconfiguration of the *Clione* swim system: Type 1A and 2A motoneurons (general excitor motoneurons), pedal serotonergic neurons, and type 12 interneurons

Stimulation of cerebral serotonergic neurons, Cr-SA and Cr-SP neurons (Figure 6A) reconfigures the *Clione* swim system by recruiting large motoneurons 1A and 2A (GEMN) motoneurons in the pedal ganglia (Arshavsky et al., 1985b; Satterlie, 1993; Norekian and Satterlie, 1996b,^2001^). These large motoneurons (80 μm diameter somas) innervate the entire parapodial muscular system—both slow-twitch fatigue resistant and fast-twitch fatigable muscle fibers—*via* the wing nerve. These large GEMNs contrast with the small motoneurons that innervate limited areas of the parapodial muscular system and only innervate the slow-twitch muscle fibers of the parapodia. Thus, when active, GEMNs increase the frequency and contractility of both the slow-twitch muscle and fast-twitch muscle fibers throughout the entire parapodia to increase the locomotor speed of *Clione* (Satterlie, 1993, 1995a).

Anterior and posterior portions of the cerebral ganglia excite the Pd-SW serotonergic neurons in the pedal ganglia and the asymmetric HE neuron in the left pedal ganglia. Additionally, Cr-V neurons weakly stimulate Pd-SW and HE neurons while inhibiting Pl-W neurons that will be described later (Figure 6A; Norekian and Satterlie, 1996b,^2001^). Both the right and the left pedal ganglia have in their antero-medial regions an agglomeration of serotonergic neurons called Pd-SW neurons (Satterlie, 1995b). Plyler and Satterlie (2020) describe each group of Pd-SW neurons as consisting of 5–9 somas that range from 20 to 80 μm with no difference in number of somas in the right and the left pedal ganglia. Furthermore, Plyler and Satterlie (2020) describe two types of Pd-SW neurons that show different innervation patterns and electrophysiological properties. These findings suggest that one group of Pd-SW neurons innervates the slow-twitch muscles fibers of the parapodia *via* the wing-nerve and plays a role in increasing locomotor speed by increasing the force of parapodial contractions during either slow swimming or fast swimming modes.

Stimulation of Cr-SA and Cr-SP evokes plateau potentials in type 12 interneurons that are in the pleural ganglia. A simultaneous recording of swim interneuron and type 12 interneuron during slow fictive swimming is shown in Figure 3. There is one type 12 interneuron soma in each of the two pleural ganglia whose axons exit each pleural ganglia *via* the pleural-pedal connective entering the ipsilateral pedal ganglia and traversing to the contralateral pedal ganglia *via* the pedal commissure. Type 12 interneurons are functionally incorporated into the *Clione* swim CPG when stimulated to activity by cerebral serotonergic neurons. Thus, the inclusion of type 12 interneurons into the CPG is one of the types of circuit-level reconfiguration that occurs to change the swimming locomotor speed in *Clione* (Figure 6A; Arshavsky et al., 1985d,^1989^; Satterlie and Norekian, 1995, 1996; Pirtle and Satterlie, 2006).

The morphology of the type 12 interneuron in relationship to the anatomy of the ganglia has permitted Pirtle and Satterlie (2006) to show the relative contribution of the type 12 interneuron to the acceleration of *Clione* swimming. In these experiments, the pleural-pedal connective was cut and the dissected preparation of *Clione* (consisting of the isolated ganglia and parapodia) was subjected to a 48-hour refrigerated (4–6°C) incubation period. Thus, these experiments effectively prevent the contribution of the type 12 interneurons from influencing the locomotor CPG and therefore swim acceleration through feedback inhibition. Pirtle and Satterlie (2006) show that the type 12 interneurons cannot, by themselves, produce the swim acceleration of *Clione*, but work simultaneously with cellular changes to swim interneurons and swim motoneurons that are produced by serotonin.

Experiments by Pirtle and Satterlie (2006) suggest that type 12 interneurons may contribute to locomotor speed in several ways. First, type 12 interneurons may help produce swimming stability by enhancing synaptic communication among the CPG interneurons. Several cellular changes occur to swim CPG interneurons. One of these cellular changes is a decrease in the duration of swim CPG interneurons, also referred to as spike narrowing (Satterlie et al., 2000). This spike narrowing is hypothesized to reduce the synaptic efficacy among the swim CPG swim interneurons. Thus, one of the possible roles of type 12 interneurons is to supply enhanced synaptic efficacy to counter the loss of such synaptic efficacy because of spike narrowing of the component swim interneurons of the *Clione* swim CPG.

Second, the feedback provided by type 12 interneurons may be a source of parapodial synchronization during fast swimming in *Clione*. As previously shown, the type 12 interneurons extend their axon through the pleural-pedal connective to the ipsilateral pedal ganglia. However, an extension of the type 12 interneuron axon also crosses to the opposite side *via* the pedal commissure to the contralateral pedal ganglia. Thus, a type 12 interneuron on one side will synaptically communicate with CPG swim interneurons found in both the right and left pedal ganglia. Therefore, the type 12 interneurons may influence the timing of swim interneuron activity by causing the right and left swim interneuron activity to better coincide in time. Furthermore, active plateau potentials of type 12 interneurons only reduce the cycle period of *Clione* CPG interneuron firing between the second to the third and between the third to the fourth cycle of the swim rhythm. Thus, the role of the type 12 interneurons may be to boost locomotor acceleration prior to the contribution of serotonergic alteration of endogenous cellular properties of the swim interneurons (Pirtle and Satterlie, 2006).

The effect of the nitrergic system on swimming locomotor regulation is shown in Figure 6B. NO has been shown (through bath application of NO donors) to activate many of the same neural elements of the swim system including swim interneurons, swim motoneurons, and Pd-SW neurons (Moroz et al., 2000; Pirtle and Satterlie, 2021). However, the effect of NO on type 12 neurons and their recruitment into the swim CPG has not been demonstrated—hence the ?s in Figure 6B. There are several locations for nitrergic neurons in the ganglia of *Clione* illustrated in Figure 4B. However, the exact nitrergic neurons that stimulate Pd-SW neurons remains unknown.

The serotonergic and nitrergic neurons accelerate *Clione* swimming locomotion. On the other hand, the Pl-W neurons (Norekian and Satterlie, 1996a), pedal swim inhibitory neuron 13 (Norekyan, 1989), and the wing retraction reflex (Huang and Satterlie, 1990), inhibit swimming locomotion (Figures 7A, B). Also shown in Figure 7A are the Cr-A neurons that promote buccal cone eversion during prey capture by inhibiting Pl-W neurons. The Pl-W neuron prevents the extrusion of the BC—appendages that engage in prey capture during fast swimming—and promote their withdrawal by stimulating Cr-B neurons (Figure 7A). Dopamine has been shown to inhibit swimming locomotion in *Clione* (Kabotyanski and Sakharov, 1988), but the only confirmed dopaminergic neuron mediating withdrawal and hence swim inhibition is cell 13 in the pedal ganglion (Figures 3D, 4C; Norekyan, 1989). Additionally, Huang and Satterlie (1990), describe a wing retraction reflex in which tactile stimuli to the parapodia evoke a parapodial (wing) retraction and inhibit swimming (Figures 3D, 7B).

Swim acceleration and whole-body withdrawal/wing retraction are competing behaviors—when one behavior is active the other behavior is suppressed. Thus, swim acceleration and whole-body withdrawal/wing retraction are said to be “mutually exclusive” (Norekian and Satterlie, 1996a). Additionally, Norekian and Satterlie (1996a) describe the *Clione* swim and passive defensive withdrawal behaviors in terms of a hierarchy in which one behavior takes precedence over the other. In their analysis, slow swimming represents the base behavior and whole-body withdrawal supersedes slow swimming. On the other hand, swim acceleration as occurs during hunting and feeding, supersedes the whole-body withdrawal behavior. Thus, the whole-body withdrawal circuit inhibits the swimming circuit during slow swimming. Contrastingly, hunting and feeding behavior excites the swim circuit while simultaneously inhibiting the whole-body withdrawal circuit. Figure 6A shows the serotonergic Cr-SV neurons inhibit the Pl-W neurons during swim acceleration (Norekian and Satterlie, 1996a,b; Satterlie and Norekian, 2001).

## Reconfiguration of the swim CPG and recruitment of neurons during the swim acceleration also activates circuits regulating the heartbeat, feeding apparatus, and tail and statomotor system

The heartbeat of *Clione* increases when tactile stimuli are applied to the tail and is directly influenced by neurons of the pedal and intestinal ganglia (Arshavsky et al., 1990; Malyshev et al., 2008). When engaged in fast swimming, a circuit of neurons favors an increase in the heart rate (Figure 8). The Cr-SA serotonergic neurons contribute to an increase in heart rate by exciting the asymmetric heart excitor (HE) neuron found in the dorsal surface of the left pedal ganglion. HE in turn stimulates contraction of the ventricle (Satterlie and Norekian, 1995; Malyshev et al., 2008). The HE neuron also stimulates the Z cell found in the left intestinal ganglia, and the Z cell subsequently stimulates auricle contraction. Another finding by Malyshev et al. (2008) is that tail stimulation excites intestinal heart excitor neurons (Int-HE neurons) and these neurons stimulate the contraction of both the ventricle and the auricle. While NO stimulates the HE neuron (Moroz et al., 2000) it is unknown which nitrergic neurons participate in this response (Figures 4B, 6B).

Decreased heart rate is favored when the Pl-W neurons of the pleural ganglion’s whole-body withdrawal system excites Int-HI neurons. The Int-HI neurons subsequently inhibit both ventricular and auricle contraction (Malyshev et al., 2008). A somewhat contradictory finding by Malyshev et al. (2008) is that the Pl-W neurons weakly stimulate Int-HE cells and Z cells. Malyshev et al. (2008) Suggest that this action of Pl-W neurons is to help prime the heart (i.e., “fill the ventricle”) so that upon resumed swimming the heart is poised to efficiently “re-inflate” the flaccid *Clione* body.

*Clione* has an open circulatory system and a hydrostatic skeleton. The increased heart rate that occurs during tail stimulation or during feeding may contribute to swim acceleration and facilitate hydraulic inflation of the BC (Arshavsky et al., 1990). Szymik and Satterlie (2017) have shown that fluid in *Clione*’s hemocoel is distributed to the parapodia and head and that constriction of the neck of *Clione* occurs during buccal cone eversion. Additionally, the distribution of hemocoelic fluid to the wings may control wing stiffness to contribute to swimming speed (Szymik and Satterlie, 2011).

The coordination of locomotor acceleration and feeding is accomplished in part by a cerebral neuron referred to as a cerebral prey capture neuron (Cr-Pc; Norekian and Satterlie, 1995) illustrated in Figures 9A, B. Cr-Pc neurons excite the swim system, heart, and neurons that control buccal cone eversion. Thus, Cr-Pc neurons coordinate locomotor and feeding circuits when *Clione* is engaged in feeding on its prey, *L. helicina*. As already mentioned, coordination of locomotion and heartbeat are important aspects of feeding behavior. In addition to activating elements of *Clione*’s swim system and the HE neuron, Cr-Pc neurons are responsible for controlling the circular smooth muscle in the BC that promote buccal cone eversion while simultaneously inhibiting neurons Cr-B and Cr-L that control buccal cone withdrawal (Figure 9A; Norekian and Satterlie, 1995). The antagonistic neurons, Cr-A and Cr-B, that produce opposite effects on the BC are also coordinated during feeding such that both neurons are activated simultaneously during the late phase of feeding behavior. This coordination is achieved by the recruitment of the GABAergic Cr-Aint neurons as illustrated in Figure 9B (Norekian and Satterlie, 1993; Norekian and Malyshev, 2006).

In the circuit illustrated in Figure 9B, GABAergic Cr-BM neurons mediate the co-activation of both Cr-A and Cr-B neurons through a buccal ganglion interneuron, Bc-PIN. The GABAergic Cr-BM neurons excite Bc-PIN interneurons that then communicate with three neurons in the cerebral ganglion that control buccal cone eversion and withdrawal—the Cr-Aint, Cr-A and Cr-B neurons. The Bc-PIN interneurons are the linkage that co-activates both Cr-A and Cr-B neurons during feeding behavior. Functionally, the recruitment of Cr-BM neurons may allow differential movement of the BC during different phases of feeding behavior (Norekian and Malyshev, 2006; Norekian et al., 2019).

During slow swimming, *Clione* maintains a relatively stable vertical position in the water column. However, during feeding *Clione* may make circular and spiraling movements as it hunts and captures its prey. Therefore, reconfiguration of the *Clione* swim system also coordinates the swim system to tail movements that control the posture of *Clione*. Control of tail movements has been described by Norekian (1995) and Panchin et al. (1995a). The circuits controlling the tail movements of *Clione* are illustrated in Figures 10A, B.

A key component of postural control in *Clione* are the paired statocysts that reside on the dorsal surface of the pedal ganglia. The statocysts have been described by Tsirulis (1974) and consist of 9–11 receptor cells (statocyst receptor cells, SRCs) forming a spherical structure with a cavity in which resides a statolith. The SRCs respond to gravity and receive differential input from CPC1 neurons located in the cerebral ganglia. Some SRCs receive excitatory input from CPC1 neurons while others receive inhibitory input from CPC1 neurons (Figure 10A). Panchin et al., 1995a,b propose that the differential inputs to SRCs from CPC1 neurons modify the output of SRCs during behavioral responses. The SRCs excite several cerebral neurons that control swimming and movement of the tail—these include the CPA1/CPB1, CPB2, and CPB3 neurons.

CPA1/CPB1 neurons are likely serotonergic (Panchin et al., 1995b) and correspond to Cr-SA and Cr-SP neurons respectively as described by Satterlie and Norekian (1995). The CPA1/CPB1 neurons excite both swim interneurons composing the swim CPG and swim motoneurons. Thus, SRCs can alter the locomotor speed of *Clione* through this part of the statocyst circuit. CPB2 neurons are excited by the SRCs and in turn excite swim motoneurons. The CPB3 neurons are excited by the SRCs and control tail movement through the excitation of tail motoneurons in the pedal ganglion (Figure 10A; Panchin et al., 1995a).

In addition to the neurons described above by Panchin et al. (1995a), there are neurons identified by Norekian (1995) that control *Clione* tail movements. These neurons are designated cerebral T neurons (Cr-T in Figure 10B). Cr-T neurons occur in pairs on the ventral side in each of the cerebral ganglia (both right and left cerebral ganglia). One member of the Cr-T pair, Cr-T1, has a soma of 80 μm and is positioned near the emergence of the cerebral commissure while the other member of the Cr-T pair, Cr-T2, has a soma of 60 μm and is positioned immediately anterior to Cr-T1. The axons of Cr-T1 and Cr-T2 neurons ultimately exit the ipsilateral pedal ganglion to innervate the ipsilateral dorsolateral tail muscles in the body wall.

Stimulation of Cr-T1 and Cr-T2 neurons produce tail bending in a coordinated fashion. When a single Cr-T1 neuron is stimulated it will produce ipsilateral bending of the tail such that the tail bends to the ipsilateral direction. However, when a left and right Cr-T1 neurons are stimulated together, the tail bends in a dorsal direction without deviating from the midline of the long body axis. There are no synaptic connections between Cr-T neurons and the neurons previously described that control buccal cone eversion (i.e., Cr-A neurons). Currently, there is also no evidence that the Cr-T neurons interact with SRCs or the other neurons described in Figure 10A. However, Cr-T neurons do monosynaptically excite swim motoneurons (both small motoneuron and large 1A/2A, general excitor motoneurons; Norekian, 1995).

Nitric oxide participates in locomotor and feeding circuits and excites the HE neuron to affect heart rate (Moroz et al., 2000). Additionally, NO may participate in the statomotor system (Panchin et al., 1995c; Moroz et al., 2000). However, the exact role that the specific nitrergic neurons play in modulation of locomotor behavior, feeding behavior, heartbeat regulation, and body orientation in *Clione* remains unclear—hence the question marks in Figure 6B that indicates that it is currently unknown what nitrergic neurons are involved. Nitrergic modulation at the circuit level may be a redundant system to that of serotonin because serotonergic neurons participate (e.g., Cr-SA and CPA1/CPB1 neurons). On the other hand, nitrergic modulation may work in separately or more subtly in combination with the serotonergic system in controlling locomotor speed, heartbeat, feeding, and body orientation at the circuit level. Further research of the circuit level role of NO is required to confirm these hypotheses.

## Cellular mechanisms and ion channels modulating *Clione* locomotor speed

Neuromodulation involves the release of substances (i.e., neuromodulators) that change ongoing activity in neural circuits such that the output of the neural circuit is changed. Neuromodulation may affect synaptic communication among neurons forming a CPG, may affect the electrical characteristics of neurons forming a CPG, or may affect both synaptic communication and electrical characteristics of neurons forming a CPG. The change in neural circuit output commonly results when the neuromodulator binds to a receptor of one or more neurons in the circuit to start a signal transduction cascade in these target neurons that ultimately change their synaptic communication, electrical properties, or both synaptic communication and electrical properties. Neuromodulation may involve enhancement or depression of the neural circuit output to adjust behavior to suit changing environmental conditions (Kaczmarek and Levitan, 1987; Marder and Thirumalai, 2002; Dickinson, 2006; Harris-Warrick, 2011; Bucher and Marder, 2013; Nadim and Bucher, 2014; Golowasch, 2019). Moreover, the complexity and diversity of neuromodulatory effects may involve interconnected elements that are extrinsic to the CPG circuitry or that are an intrinsic part of the CPG circuitry (Katz, 1998).

In *Clione*, serotonin and NO are two neuromodulator substances that enhance swim CPG output and thus contribute to locomotor acceleration. Both serotonin and NO are extrinsic to the *Clione* swim CPG. Furthermore, both serotonin and NO modulate *Clione* swimming locomotion by eliciting changes in swim interneurons comprising the *Clione* swim CPG circuit (Satterlie and Norekian, 1995; Moroz et al., 2000; Satterlie et al., 2000). Nitric oxide’s ability to alter *Clione* swimming locomotion is mediated by soluble guanylyl cyclase (sGC), the production of cyclic guanosine monophosphate (cGMP) by sGC, and the activation of cGMP-dependent protein kinase activity (Pirtle and Satterlie, 2021). Less is known about the signal transduction mechanisms that mediate the similar acceleration of swimming locomotion by serotonin. However, Pirtle and Satterlie (2021) have shown that the ability of serotonin to increase *Clione* locomotor speed is not dependent on cGMP while the effect of NO on increasing locomotor speed is dependent on cGMP.

Both serotonin and NO-cGMP signaling cause the acceleration of *Clione* swimming through a common set of cellular mechanisms that involve modification of the electrical properties of swim interneurons. These cellular mechanisms include (1) spike-narrowing, (2) baseline depolarization, (3) an enhanced sag potential, and (4) enhanced postinhibitory rebound (Satterlie and Norekian, 1995, 2001; Satterlie et al., 2000; Pirtle and Satterlie, 2021). These cellular mechanisms are dependent on the involvement of ion channels and their corresponding ion conductance. Knowledge of ion channel operation within CPG circuits is essential for understanding how CPGs create rhythmic output and how this rhythmic output is modulated (Grillner, 1999; Brocard, 2019). The ion channels that are involved in the *Clione* swim CPG have been described with some difficulty.

**FIGURE 11:**
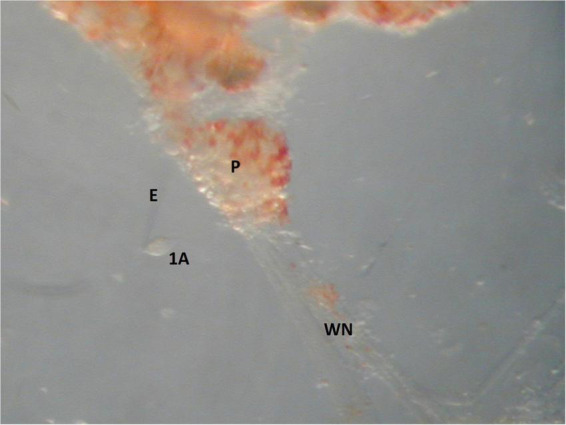
An image of the cell isolation method used in single electrode voltage clamp experiments. The soma of a large motoneuron, 1A, is isolated by the recording electrode E. The width of the 1A motoneuron is 30 μm and the width of the pedal ganglia, P, is 350 μm.

The ionic currents characteristic of *Clione* swim CPG interneurons is challenging to study for several reasons. First, these swim interneurons must be found electrophysiologically by impaling them with microelectrodes. A second problem arises because the electrical coupling between other synergistic swim interneurons and between synergistic motoneurons creates space clamp issues when recording membrane currents of swim interneurons in single electrode voltage clamp experiments. To overcome the space clamp problem, an electrically identified swim interneuron must be physically isolated by using the recording electrode to pull the identified swim CPG interneuron out of the pedal ganglia. This isolation process is done by slowly moving the microelectrode impaling the swim CPG interneuron *via* a micromanipulator to gradually wrench the interneuron soma away from surrounding cells within the ganglia without rupturing its plasma membrane (Figure 11).

**FIGURE 12:**
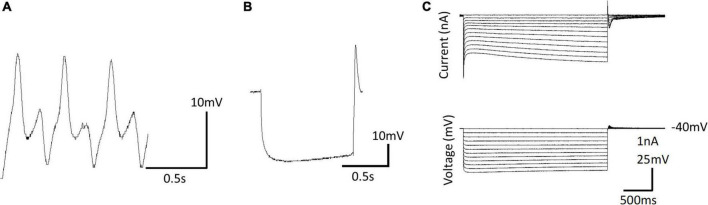
**(A)** Intracellular recording of a dorsal swim interneuron (type 7 swim interneuron) before physically isolating the cell. **(B)** Postinhibitory rebound and sag potential evoked by injecting a –1 nA, 1 s duration current pulse into the isolated cell identified in **A**. **(C)** Single electrode voltage clamp of the isolated cell **(A)** showing the development of slow inward current evoked by hyperpolarizing voltage steps from a holding potential of –40 mV—the hyperpolarization-activated cyclic nucleotide-gated inward current, I_h_.

Ionic currents have been characterized in *Clione* swim interneurons using this method of physically isolating the swim interneuron in single-electrode voltage clamp experiments (Pirtle and Satterlie, 2004). Figure 12A shows the intracellular recording of a type 7 swim interneuron before isolation. One current of particular interest is the hyperpolarization-activated cyclic nucleotide-gated cation current, I_h_, mediated by HCN ion channels). I_h_ is evoked in single electrode voltage clamp experiments with hyperpolarizing voltage steps that are applied from a holding potential of −40 mV. I_h_ is characterized by a slowly developing inward current that is mediated by an increase in both Na^+^ and K^+^ conductance (Pape, 1996; Lüthi and McCormick, 1998). In intracellular—or current clamp—recordings, the I_h_ current manifests as a slow depolarizing drift in the membrane potential during the application of hyperpolarizing current injection. This slow depolarizing drift in membrane potential is called a sag potential. Much of the information about I_h_ in *Clione* swim interneurons is inferred from changes in the sag potential produced by applying hyperpolarizing current to these cells during the intracellular recording of membrane potential in these cells (Figures 12B, C; Pirtle et al., 2010; Pirtle and Satterlie, 2021).

Intracellular recording of the sag potential requires isolating cells, not because of space clamp issues as associated with single electrode voltage clamp, but because these cells receive synaptic input that interferes with the recording of individual cellular properties of swim interneurons. Synaptic blockade using the muscarinic acetylcholine receptor antagonists, atropine, the glutamate receptor antagonist, 6-cyano-7-nitroquinoxaline-2,3-dione disodium (CNQX), and the sodium ion channel blocker, tetrodotoxin is used to thus chemically isolate *Clione* swim interneurons for intracellular recordings (Pirtle and Satterlie, 2004). The sag potential of swim interneurons can change with the application of the neuromodulator serotonin and the second messenger, cGMP. The addition of serotonin or cGMP increases fictive swim frequency as recorded intracellular from *Clione* swim CPG interneurons. Furthermore, serotonin and cGMP enhance the sag potential of *Clione* swim CPG interneurons by making the trajectory of the sag potential reach a more depolarized level faster. Thus, the enhancement of the sag contributes to increased frequency of swim interneuron action potentials and therefore an increase of the locomotor speed of *Clione* (Pirtle et al., 2010; Pirtle and Satterlie, 2021).

Because both serotonin and NO contribute to the enhancement of the sag potential, both serotonergic and nitrergic modulatory inputs converge to affect the activity of a common ion channel—the HCN ion channel that produces I_h_ and corresponding sag potential—in the same swim interneurons comprising the *Clione* swim CPG. In this respect, the *Clione* swim CPG is like the noradrenergic and serotonergic modulation of I_h_ in thalamic relay neurons where both noradrenalin and serotonin enhance I_h_ (McCormick and Pape, 1990). Kintos et al. (2016) indicate that the “functional consequences” of the convergence are “less clear.” Kintos et al. (2016) use a model of the crab gastric mill to demonstrate that the convergence of two neuromodulators onto a single ion channel is not merely redundant but may produce “distinct effects.” It is possible that the serotonergic and nitrergic modulation of the *Clione* swim CPG are not just redundant systems that affect the locomotor speed in the same way—or additive way. Rather, it may be that serotonergic and nitrergic effects are subtly different in their effects.

It is known with certainty that NO produces swim acceleration in *Clione* through cGMP-mediated changes in the cellular properties of neurons forming the *Clione* swim locomotor CPG (Moroz et al., 2000; Pirtle and Satterlie, 2021). However, it is not known what signal transduction mechanism mediates the serotonergic modulation of *Clione* swimming speed. One possible candidate is that serotonin binds to a G-protein coupled receptor that starts a cAMP signal transduction cascade. This is a likely candidate because both cAMP (through binding of epinephrine to beta-1 adrenergic receptors and subsequent activation of adenylyl cyclase) and cGMP (through NO stimulation of sGC) modulate I_f_—I_h_ associated with the vertebrate sinoatrial node pacemaker cells (Musialek et al., 1997; Accili et al., 2002; Biel et al., 2009; Hennis et al., 2022) by binding to the intracellular C-terminal cyclic nucleotide binding domain (CNBD) of the HCN channels to promote their opening (Biel et al., 2002; Hennis et al., 2022). While the binding of cAMP to the CNBD is greater than cGMP in mammalian HCN channels, the effect of both cAMP and cGMP is to increase heart rate (Accili et al., 2002; Craven and Zagotta, 2006). Additionally, the crayfish swimmeret motoneurons respond to both cAMP and cGMP in cooperative way to increase the frequency of rhythmic bursting in these motoneurons. In the crayfish swimmeret system, cAMP increases locomotor frequency while cGMP (mediated by NO) is facilitatory (Mita et al., 2014). Similar signal transduction mechanisms involving cAMP and cGMP may function in *Clione* swim interneurons to modulate swimming locomotor speed through an increase in CPG interneuron excitability.

There are several metabotropic 5-HT receptor subtypes in invertebrates, including mollusks (Tierney, 2001) and some of these molluscan 5-HT receptors are linked to cAMP production *via* activation of adenylyl cyclase (Cohen et al., 2003). However, preliminary experimental results indicate that cAMP inhibits swimming locomotion in *Clione*. When the membrane permeable cAMP analog, 8-bromo-cAMP is administered, a decrease in fictive locomotor activity is recorded from *Clione* swim interneurons (Pirtle, unpublished data). Thus, cAMP does not contribute to swimming acceleration in *Clione*, but rather inhibits swimming. These unpublished data are consistent with the findings that the HCN ion channel cloned from the closely related marine mollusk, *Aplysia californica*, is more sensitive to cGMP than to cAMP (Yang et al., 2015) and that in *Clione*, the CNBD of HCN channels may preferentially bind cGMP. Cyclic AMP also disrupts swimming locomotion in the escape swim CPG of *Tritonia* (Clemens et al., 2007). Clemens et al. (2007) hypothesize that fluctuating concentrations of cAMP and intracellular Ca^2+^ may be involved in controlling the timing of the swimming activity in *Tritonia*—especially the termination of swimming. Levels of cGMP and cAMP in *Clione* swim interneurons may work similarly. In *Clione*, cGMP works as an accelerator (Pirtle and Satterlie, 2021) and cAMP (unpublished data) works as a brake. The balance of cGMP and cAMP may be critical for determining the frequency of action potentials generated in *Clione* swim interneurons and thus influence locomotor speed.

## Discussion

Modulation of swimming behavior in *Clione* involves both circuit-level and cellular changes. At the circuit level, recruitment of interneurons (i.e., type 12 interneurons) to reconfigure the swim CPG and recruitment of motoneurons (i.e., general excitor motoneurons—or 1A and 2A motoneurons and Pd-SW neurons) are essential components to *Clione* swim acceleration. Swimming in *Clione* involves a two-geared system and modulation of swimming speed can occur by a change of gears or a change within gears—the former requiring a change in the frequency output of the *Clione* swim CPG and the later requiring changes in muscle contractility (Satterlie et al., 1990; Satterlie, 1991, 1993; Plyler and Satterlie, 2020). Cellular changes that occur during swim acceleration addressed in this review focus on how the hyperpolarization-activated cyclic nucleotide channel current, I_h_, contributes to swim acceleration in *Clione*.

Experiments by Pirtle and Satterlie (2006) suggest that the role of type 12 interneuron may involve more than providing feedback inhibition as originally suggested (Arshavsky et al., 1985d,1993a). The involvement of type 12 interneurons to synaptic efficacy and synchronization of the locomotor appendages, the parapodia, represent critical gaps in the current understanding of how an interneuron’s recruitment into a CPG contributes to reconfiguration locomotor output (Pirtle and Satterlie, 2006). Additionally, while it is known that serotonin recruits *Clione* type 12 interneurons it is not known how NO affects these cells. Additional, experiments to identify how NO may contribute to type 12 interneuron recruitment are necessary.

There are several unanswered questions regarding motoneuron recruitment in *Clione*—particularly the recruitment of Pd-SW cells. It is clear that one group of Pd-SW cells is responsible for increasing the strength of parapodial movement through the modulation of slow-twitch muscle fibers (Satterlie, 1995b; Plyler and Satterlie, 2020). However, the role of Pd-SW neurons in modulating *Clione* swimming speed within the slow swimming gear is unclear. Furthermore, the contribution of one group of Pd-SW cells that are hypothesized to control the body wall musculature during fast swimming remains unresolved primarily due to the lack of morphological data on the distribution of these neurons to the body wall. The Pd-SW neurons to the body wall may work in concert with serotonergic modulation of heartbeat to distribute hemolymph to the wings resulting in enhanced wing stiffness as a mechanism to increase locomotor speed (Arshavsky et al., 1990; Szymik and Satterlie, 2017; Plyler and Satterlie, 2020). Because NO also modulates both locomotor behavior and heartbeat in *Clione* (Moroz et al., 2000; Pirtle and Satterlie, 2021), the coordination and relative contribution of serotonergic and nitrergic systems in controlling this possible mechanism requires attention.

At the cellular level, the HCN ion channels contribute to modulation of swimming in *Clione*. HCN ion channels produce the I_h_ current and corresponding sag potential. Blocking I_h_ with ZD7288 slows but does not completely abolish swimming in *Clione*. However, blocking I_h_ with ZD7288 does prevent serotonin-induced swim acceleration in *Clione* (Pirtle et al., 2010). Therefore, I_h_ is a modulatory target for serotonin but is not essential for rhythm generation. I_h_ is also a target for NO-cGMP modulation of *Clione* swimming (Pirtle and Satterlie, 2021). Other possible roles for I_h_ may include modulation of synaptic communication. I_h_ has been shown by Yang et al. (2015) to be found in siphon motoneurons of *Aplysia*, and in this regard, I_h_ contributes to classical conditioning of the siphon withdrawal reflex through a NO signaling pathway and synaptic facilitation. Additionally, I_h_ has been shown to modulate synaptic transmission and synaptic strength through serotonin-induced cAMP production in the neuromuscular junction of crayfish (Beaumont and Zucker, 2000). Therefore, there may be a similar role of I_h_ in *Clione*. I_h_ in *Clione* swim interneurons may respond to a NO-cGMP signal to enhance transmitter release during fast swimming speed. This possible mechanism may work in concert with type 12 interneuron recruitment to help strengthen synaptic communication to oppose the decreased synaptic efficacy associated with swim interneuron spike narrowing during swim acceleration (Satterlie et al., 2000; Pirtle and Satterlie, 2007).

Finally, the effect of both serotonin and NO on swim acceleration are similar—both serotonergic and nitrergic modulatory systems accelerate *Clione* swimming through common mechanisms that involve I_h_ (Satterlie and Norekian, 1996; Satterlie et al., 2000; Pirtle et al., 2010; Pirtle and Satterlie, 2021). Currently, the signal transduction mechanism and second messengers that mediate the serotonergic response in *Clione* swim interneurons is unknown. Serotonin and NO may be redundant neuromodulators that work separately or together. Additionally, there may be cross communication between serotonergic and nitrergic neuromodulatory systems, however, there may also be subtle differences in the modulatory effects of serotonergic and nitrergic modulation in the *Clione* swim system. Identification of the second messengers that mediate serotonergic modulation of *Clione* will be essential to elucidate these possibilities.

The modulation of the *Clione* swim CPG shares many common features with that of other locomotor systems. Two modulatory paradigms that function in the *Clione* swim CPG are (1) motoneuron and interneuron recruitment and (2) modification of cellular properties and ion channels. Further research of the *Clione* swim system may reveal more fundamental similarities and differences with other invertebrate and vertebrate locomotor CPGs. An important part of the locomotor control mechanism in *Clione* that needs further clarification is the role of second messengers. Signal transduction involving second messengers is critical for determining how locomotor CPG circuits are modulated (Clemens and Katz, 2001; Clemens et al., 2007). A complete analysis of the role of cyclic nucleotides in changing locomotor speed in *Clione* promises to yield significant information regarding how locomotor CPGs are modulated.

The evolution of locomotor activity in *Clione* in relationship to other marine gastropod mollusks should be addressed. Several other molluscan species exhibit locomotor activity that has evolved repeatedly and involves the gastropod foot. In *Clione*, the parapodia are derived from the foot and it is the dorsal-ventral flexion of the parapodia that propel *Clione* in swimming locomotion. In other marine gastropods, the dorsal-ventral bending of the body wall (e.g., *Tritonia* and *Pleurobranchaea*) and lateral bending of the body wall (e.g., *Melibe*) produces swimming locomotor activity and, in both instances, is associated with muscles of the foot. The differences in locomotor abilities among the various marine gastropods likely reflects adaptive radiation as the different species occupy different ecological niches. For example, *Clione* is holoplanktonic and is typically in a state of continuous swimming to maintain its position in the water column. This contrast with, for example, the benthic *Tritonia* that crawls on the ocean bottom and only swims as a means of predatory avoidance (Katz et al., 2001; Willows, 2001). Despite the different modes of swimming among the different marine gastropods, the neural circuitry in each share a common ancestry. For example, the cerebral serotonergic neurons, the *Clione* Cr-SP group, are homologs of the *Tritonia* serotonergic dorsal swim interneurons (DSIs). One of the unique differences in evolution that has occurred is the different roles that these homologous neurons take in terms of swimming behavior. In *Tritonia*, the serotonergic DSIs are both part of the *Tritonia* CPG and modulate swimming behavior. The DSIs are therefore considered to participate in intrinsic neuromodulation of *Tritonia* swim behavior. In *Clione*, these homologs, the Cr-SP neurons, are extrinsic neuromodulators (Katz et al., 2001). These evolutionary differences, as previously stated, are principally because *Tritonia* and *Clione* locomotor behavior are specific for the ecological niche that these two species occupy.

Additionally, the closely related *Clione antarctica* of the southern hemisphere shows significant differences in its locomotor system when compared to the northern hemisphere congener, *C. limacina* (Rosenthal et al., 2009; Dymowska et al., 2012). Foremost of the differences between *C*. *antarctica* and *C*. *limacina* is the absence of fast swimming in *C*. *antarctica* and associated loss of the neural circuitry (1A and 2A motoneurons) and fast-twitch musculature that produces fast swimming. Rosenthal et al. (2009) suggest that the loss of fast swimming in *C*. *antarctica* is an evolutionary adaptive tradeoff in which the species lost locomotor acceleration in favor of increased aerobic metabolism in response to living in a much colder environment. The evolutionary differences of *C*. *limacina* and *C*. *antarctica* evoke important questions. For example, both *C*. *limacina* and *C*. *antarctica* are feeding specialist that prey on *L. helicina* (Dymowska et al., 2012). The neural circuitry underlying locomotion, feeding, and statomotor systems are linked in *C*. *limacina*. The connections, or lack thereof, between locomotor, feeding, and statomotor systems in *C*. *antarctica* remain unknown. Additionally, similarities and differences in the neuromodulatory serotonergic and nitrergic systems between *C*. *limacina* and *C*. *antarctica* need to elucidated.

## Author contributions

The author confirms being the sole contributor of this work and has approved it for publication.
